# Alcohol Consumption and Risk of Parkinson's Disease: Data From a Large Prospective European Cohort

**DOI:** 10.1002/mds.28039

**Published:** 2020-05-01

**Authors:** Susan Peters, Valentina Gallo, Paolo Vineis, Lefkos T. Middleton, Lars Forsgren, Carlotta Sacerdote, Sabina Sieri, Andreas Kyrozis, María‐Dolores Chirlaque, Raul Zamora‐Ros, Oskar Hansson, Jesper Petersson, Verena Katzke, Tilman Kühn, Olatz Mokoroa, Giovanna Masala, Eva Ardanaz, Salvatore Panico, Manuela M. Bergmann, Timothy J. Key, Elisabete Weiderpass, Pietro Ferrari, Roel Vermeulen

**Affiliations:** ^1^ Institute for Risk Assessment Sciences Utrecht University Utrecht The Netherlands; ^2^ Department of Neurology University Medical Center Utrecht Utrecht The Netherlands; ^3^ Centre for Primary Care and Public Health Queen Mary University of London London UK; ^4^ School of Public Health Imperial College London London UK; ^5^ Department of Clinical Sciences, Neurosciences Umeå University Umeå Sweden; ^6^ Unit of Cancer Epidemiology Città della Salute e della Scienza University‐Hospital Turin Italy; ^7^ Center for Cancer Prevention Turin Italy; ^8^ Epidemiology and Prevention Unit, Fondazione Istitutodi Ricovero e Cura a Carattere Scientifico (IRCCS) Istituto Nazionale dei Tumori Milan Italy; ^9^ Hellenic Health Foundation Athens Greece; ^10^ First Department of Neurology National and Kapodistrian University of Athens Athens Greece; ^11^ Department of Epidemiology Regional Health Council, Instituto Murciano de Investigación Biosanitaria (IMIB)‐Arrixaca Murcia Spain; ^12^ Centrode Investigación Biomédica en Red (CIBER) in Epidemiology and Public Health Madrid Spain; ^13^ Department of Health and Social Sciences, Universidad de Murcia Murcia Spain; ^14^ Unit of Nutrition and Cancer, Epidemiology Research Program Catalan Institute of Oncology, Bellvitge Biomedical Research Institute, Hospitalet de Llobregat Barcelona Spain; ^15^ Clinical Memory Research Unit, Department of Clinical Sciences Lund University Malmö Sweden; ^16^ Memory Clinic Skåne University Hospital Lund Sweden; ^17^ Department of Neurology, Skåne University Hospital Lund University Malmö Sweden; ^18^ German Cancer Research Centre Heidelberg Germany; ^19^ Public Health Division of Gipuzkoa BioDonostia Research Institute San Sebastian Spain; ^20^ Cancer Risk Factors and Life‐Style Epidemiology Unit Institute for Cancer Research, Prevention and Clinical Network–Cancer Research and Prevention Institute (ISPRO) Florence Italy; ^21^ Navarra Public Health Institute Pamplona Spain; ^22^ Institutode Investigación Sanitaria de Navarra (IdiSNA) Navarra Institute for Health Research Pamplona Spain; ^23^ Dipartimento di Medicina Clinica e Chirurgia Federico II University Naples Naples Italy; ^24^ German Institute of Human Nutrition Potsdam‐Rehbrücke Germany; ^25^ Nuffield Department of Population Health University of Oxford Oxford UK; ^26^ International Agency for Research on Cancer Lyon France; ^27^ Julius Center for Health Sciences and Primary Care University Medical Center Utrecht Utrecht The Netherlands

**Keywords:** alcohol, EPIC, epidemiology, Parkinson, prospective cohort

## Abstract

**Background:**

Parkinson's disease (PD) etiology is not well understood. Reported inverse associations with smoking and coffee consumption prompted the investigation of alcohol consumption as a risk factor, for which evidence is inconclusive.

**Objective:**

To assess the associations between alcohol consumption and PD risk.

**Methods:**

Within NeuroEPIC4PD, a prospective European population‐based cohort, 694 incident PD cases were ascertained from 209,998 PD‐free participants. Average alcohol consumption at different time points was self‐reported at recruitment. Cox regression hazard ratios were estimated for alcohol consumption and PD occurrence.

**Results:**

No associations between baseline or lifetime total alcohol consumption and PD risk were observed. Men with moderate lifetime consumption (5–29.9 g/day) were at ~50% higher risk compared with light consumption (0.1–4.9 g/day), but no linear exposure–response trend was observed. Analyses by beverage type also revealed no associations with PD.

**Conclusion:**

Our data reinforce previous findings from prospective studies showing no association between alcohol consumption and PD risk. © 2020 The Authors. *Movement Disorders* published by Wiley Periodicals, Inc. on behalf of International Parkinson and Movement Disorder Society.

The etiology of Parkinson's disease (PD) is complex and likely involves both genetic and environmental factors.^1^ There are strong and consistent observations that cigarette smoking^2^, ^3^, ^4^ and coffee drinking^4^, ^5^ are associated with a decreased risk of PD. Although the specific mechanisms are still poorly understood, these observed associations are probably not explained by reverse causation or confounding.^3^, ^4^, ^5^

In addition to smoking and coffee consumption, alcohol consumption is another possible factor involved in the development of PD. Several meta‐analyses on the association between alcohol consumption and the risk of PD have been conducted, all suggesting an inverse association.^6^, ^7^ The results, however, are as yet inconclusive: the inverse association was mainly observed in retrospective case‐control studies, but was not as clear in studies based on prospective cohorts.

By design, case‐control studies have some limitations. First, these studies are prone to recall bias, as the disease status and disease characteristics may affect the retrospective assessment of alcohol consumption habits. Another risk is selection bias because controls may not well reflect the source population. Finally, associations observed in case‐control studies may be the result of reverse causality, for example, when premorbid changes led to reduced alcohol consumption.

Few large prospective studies, largely avoiding these biases, have been published on alcohol consumption and PD risk. Three large‐scale cohort studies, all conducted in the United States, concluded that there is no or only weak evidence for a decreased risk of PD in association with total alcohol consumption.^8^, ^9^, ^10^ Specific types of alcoholic beverages, however, were suggested to have different effects: a lower PD risk was reported for moderate beer drinkers,^8^, ^9^ whereas liquor consumption was associated with higher risk.^8^

Our objective was to assess the association between alcohol consumption and the risk of PD in a large European prospective cohort study. We present risk estimates for average alcohol consumption 12 months prior to the recruitment (short term) and during lifetime, that is, since the age of 20 years (long term), as well as the PD risks associated with different types of alcoholic beverages.

## Methods

In the early 1990s, the European Prospective Investigation into Cancer and Nutrition (EPIC) cohort study has been established, with more than 521,000 participants.^11^ At recruitment, the participants were mainly between 35 and 70 years old and lifestyle factors were self‐reported using validated questionnaires. Ethical approval was obtained from the ethical committee of the International Agency for Research on Cancer and ethical review boards of each participating center. All participants signed an informed consent. NeuroEPIC4PD comprises a subset of 220,494 participants within EPIC, recruited in Germany, Greece, Italy, the Netherlands, Spain, Sweden, and the United Kingdom.^12^

### Case Ascertainment

In NeuroEPIC4PD, 881 PD cases have been identified and their diagnosis has been validated through clinical record review.^12^ We limited our analyses to 209,998 participants, including 694 incident PD cases, after removing 34 cases without date of diagnosis, 122 prevalent cases, 212 participants with PD‐like conditions, and 10,128 participants (including 31 PD cases) with missing information on alcohol consumption or smoking status at baseline.

### Assessment of Alcohol Consumption

Average consumption of alcoholic beverages during the 12‐month period before recruitment and at ages 20, 30, 40, and 50 years was collected via validated country‐specific dietary and standardized lifestyle questionnaires. Alcohol consumption at each point in time was derived from the consumption frequency of glasses of beer, cider, wine, fortified wine, sweet liquor, or distilled spirit. Total alcohol intake was expressed as grams per day (g/day) based on country‐specific and sex‐specific standard glass volumes and beverage‐specific ethanol percentages derived from 24‐hour dietary recalls conducted in a 10% subsample of the EPIC cohort. A more detailed description of the variables can be found elsewhere.^13^ Information on lifetime alcohol was available for 150,197 participants (including 478 incident PD cases) because these data were not collected in Sweden and Naples (Italy).

Alcohol consumption was categorized into <0.1 g/day (at recruitment, nonconsumers; at lifetime, never consumers), 0.1 to 4.9 g/day (reference category), 5.0 to 14.9 g/day, 15.0 to 29.9 g/day, 30.0 to 59.9 g/day, and ≥ 60 g/day. As per previous EPIC papers,^14^, ^15^, ^16^ we used light consumers (0.1–4.9 g/day) as the reference category because total abstainers may represent a highly selective group. For lifetime consumption of specific types of alcoholic beverages, including beer, wine, fortified wine, and spirit/liquor, ≥15 g/day was the highest category.

### Statistical Analyses

Cox regression models using age as the underlying time variable were applied to investigate the effects of alcohol consumption on the risk of PD. Models were adjusted for sex, age at recruitment, country, smoking status at recruitment (never, former, current smoker) and average coffee consumption at recruitment (never, >0–<100, 100–<250, 250–<500, >500 mL/day). We also considered educational level as possible confounding factor, but this variable did not modify the risk estimates (*P* > 0.1) and was therefore not included in the final models. Although other environmental factors have been reported to affect PD risk,^1^ our data did not allow for considering additional adjustments. Models were run for both alcohol consumption at recruitment and during lifetime and by type of alcoholic beverage.

We stratified analyses by sex to assess possible different associations among men and women.^17^ For sensitivity analyses, we stratified analyses by smoking status at recruitment (ever vs. never smoker) because of its strong inverse association with PD^3^ and its relation with alcohol consumption. We also tested if there was an interaction between smoking and alcohol consumption. In addition, we ran analyses separately for PD cases who were diagnosed within or after the mean of 8 years since recruitment to assess the possible effects of changes due to early disease processes. To further explore possible reverse causation as the explanation of positive findings in previous case‐control studies, we also ran the same analyses on prevalent PD cases within NeuroEPIC4PD (n = 92 with information on alcohol consumption at recruitment).

## Results

Demographic characteristics and alcohol consumption for PD cases and participants without PD in the NeuroEPIC4PD cohort are described in Table 1.

**Table 1:** Demographics and alcohol consumption habits among NeuroEPIC4PD participants with and without PD

Characteristic	NeuroEPIC4PD, N = 209,998	
	PD cases, N = 694	Cohort, N = 209,304
Sex		
Male	353, 51%	78,042, 37%
Female	341, 49%	131,262, 63%
Age at recruitment, mean (SD)	61.2 (8.2)	52.9 (9.9)
Age at diagnosis, mean (SD)	68.7 (7.9)	–
Years between recruitment and diagnosis, mean (SD)	7.9 (4.2)	–
Country		
Italy	64, 9.2%	40,111, 19%
Spain	105, 15%	24,852, 12%
United Kingdom	171, 25%	23,227, 11%
The Netherlands	13, 1.9%	16,814, 8.0%
Greece	92, 13%	25,762, 12%
Germany	50, 7.2%	25,389, 12%
Sweden	199, 29%	53,149, 25%
Alcohol consumption at recruitment		
Nonconsumer	150, 22%	37,662, 18%
Total g/day, mean (5th–95th percentile)	10 (0–47)	11 (0–47)
Beer g/day, mean (5th–95th percentile)	2.2 (0–8.6)	2.4 (0–11)
Wine g/day, mean (5th–95th percentile)	6.1 (0–31)	6.9 (0–35)
Fortified wine g/day, mean (5th–95th percentile)	0.6 (0–3.0)	0.6 (0–3.3)
Spirits g/day, mean (5th–95th percentile)	1.4 (0–7.8)	1.4 (0–7.1)
Average lifetime alcohol consumption^a^		
Never consumer	48, 10%	18 192, 12%
Total g/day, mean (5th–95th percentile)	16 (0.6–57)	16 (0.3‐60)
Beer g/day, mean (5th–95th percentile)	2.9 (0–13)	3.4 (0–17)
Wine g/day, mean (5th–95th percentile)	9.3 (0–42)	8.9 (0–39)
Fortified wine g/day, mean (5th–95th percentile)	0.8 (0–3.7)	0.8 (0–3.4)
Spirits g/day, mean (5th–95th percentile)	3.1 (0–11)	3.1 (0–14)
Smoking status at recruitment		
Never	395, 57%	100,080, 48%
Former	219, 32%	57,899, 28%
Current	80, 12%	51,325, 25%
Coffee consumption at recruitment		
Nonconsumer	79, 11%	14,500, 6.9%
>0 to <100 mL/day	154, 22%	43,560, 21%
100 to <250 mL/day	182, 26%	53,750, 26%
250 to <500 mL/day	160, 23%	48,486, 23%
≥500 mL/day	119, 17%	49,008, 23%

a Information on lifetime alcohol consumption was missing for 216 PD cases and 59,585 participants without PD.

NeuroEPIC4PD, is the study on Parkinson’s disease case ascertainment in the EPIC cohort; PD, Parkinson's disease; SD, standard deviation.

No association between alcohol consumption at recruitment and the risk of PD was observed overall nor when stratified by sex (Table 2). The average lifetime alcohol consumption also did not show an association with PD risk overall. Analyses limited to men showed increased risks for the lifetime moderate consumers (hazard ratio = 1.58 [95% confidence interval, 1.07–2.33] for 5–14.9 g/day and hazard ratio = 1.52 [95% confidence interval, 1.00–2.33] for 15–29.9 g/day) compared with light consumers (0.1–4.9 g/day), but there was no exposure–response trend (*P* = 0.55).

**Table 2:** Number of PD cases and hazard ratios by levels of alcohol consumption (g/day): consumption at recruitment, average lifetime consumption, and average lifetime consumption per type of alcoholic beverage

Alcohol consumption at recruitment (g/day)	All, n = 209,998	Men, n = 78,395		Women, n = 131,603		
	PD cases	HR^a^ (95% CI)	PD cases	HR^a^ (95% CI)	PD cases	HR^a^ (95% CI)
Nonconsumer	150	0.99 (0.80–1.24)	47	1.11 (0.77–1.60)	103	1.00 (0.76–1.32)
0.1–4.9	210	1.00 (ref)	86	1.00 (ref)	124	1.00 (ref)
5.0–14.9	174	0.95 (0.78–1.17)	91	1.01 (0.75–1.37)	83	0.96 (0.72–1.28)
15–29.9	95	1.03 (0.80–1.33)	69	1.12 (0.80–1.55)	26	1.09 (0.71–1.68)
30–59.9	53	1.05 (0.76–1.45)	48	1.22 (0.84–1.77)	5	0.85 (0.34–2.09)
≥60	12	0.69 (0.38–1.26)	12	0.81 (0.43–1.53)	0	–
*P* value for trend^b^		0.47		0.98		0.34

Average lifetime alcohol consumption (g/day)	All, n = 150,197		Men, n = 54,633		Women, n = 95,564	
	PD cases	HR^a^ (95% CI)	PD cases	HR^a^ (95% CI)	PD cases	HR^a^ (95% CI)
Never consumer	48	0.91 (0.65–1.27)	8	1.29 (0.60–2.78)	40	0.79 (0.54–1.15)
0.1–4.9	146	1.00 (ref)	40	1.00 (ref)	106	1.00 (ref)
5.0–14.9	142	1.23 (0.97–1.57)	84	1.58 (1.07–2.33)	58	1.07 (0.77–1.48)
15–29.9	77	1.07 (0.78–1.45)	64	1.52 (1.00–2.33)	13	0.82 (0.46–1.49)
30–59.9	47	0.98 (0.67–1.43)	47	1.44 (0.91–2.28)	0	–
≥60	18	0.72 (0.43–1.23)	18	1.11 (0.61–2.03)	0	–
*P* value for trend^b^		0.10		0.55		0.16

Average lifetime alcohol consumption (g/day)	Beer		Wine		Fortified wine		Spirit/liquor	
	PD cases	HR^a^ (95% CI)	PD cases	HR^a^ (95% CI)	PD cases	HR^a^ (95% CI)	PD cases	HR^a^ (95% CI)
Never consumer	177	0.94 (0.75–1.16)	103	0.95 (0.74–1.21)	251	0.79 (0.63–0.96)	220	1.16 (0.94–1.45)
0.1–4.9	234	1.00	202	1.00	211	1.00	194	1.00
5.0–14.9	48	0.94 (0.68–1.31)	87	1.15 (0.88–1.49)	13	0.97 (0.54–1.73)	49	0.99 (0.72–1.37)
≥15	19	0.85 (0.52–1.39)	86	0.94 (0.69–1.28)	3	0.91 (0.28–2.92)	15	0.72 (0.42–1.23)
*P* value for trend^2^	0.45		0.19		0.27		0.67	

a HR, adjusted for age at recruitment, sex (in combined analyses), country, smoking status, and coffee consumption; and 95% CI.

b Trend among alcohol consumers only.

PD, Parkinson's disease; HR, hazard ratio; CI, confidence interval; ref, reference.

Analyses for lifetime consumption by type of alcoholic beverage did not reveal any association with PD risk (Table 2). Stratification by smoking indicated no association between average lifetime alcohol consumption and PD risk among never smokers (Supplemental Table S1). Among ever smokers, there was a possible decreasing risk of PD with increased average lifetime alcohol consumption (*P*_trend_ = 0.07). The *P* value for interaction between lifetime average number of cigarettes per day and alcohol consumption was 0.09.

Analyses separating PD cases diagnosed within or after 8 years of recruitment revealed comparable results (data not shown). A negative exposure‐response trend between lifetime alcohol consumption and PD risk (*P* = 0.04) was observed for prevalent cases (Supplemental Table S2).

## Discussion

We observed no associations between baseline or lifetime alcohol consumption and the risk of PD in the NeuroEPIC4PD cohort. These findings are consistent with previous large prospective studies.^8^, ^9^, ^10^ Our analyses by type of alcoholic beverage (beer, wine, fortified wine, spirit/liquor) also revealed no associations.

In contrast to smoking and coffee consumption, for which inverse associations with PD risk have been consistently reported by several groups across study designs,^3^, ^4^, ^5^ prospective studies on alcohol point toward no association. The observed inverse associations with alcohol reported in PD case‐control studies has been suggested to be related to recall bias, reverse causation, or residual confounding by smoking.^9^, ^10^

Reverse causation can be the result of disease‐related changes in behavior, for example, when PD patients were more prone to stop or reduce drinking because of their symptoms.^10^ Patients may recall their previous drinking habits differently because of these changes. This possibility is supported by our sensitivity analyses among prevalent PD cases, mimicking a case‐control study where cases are typically interviewed after diagnosis, which would have led to a different conclusion. Although based on much fewer cases, a decreasing risk of PD was observed with lifetime alcohol consumption (*P*_trend_ = 0.04). Because no association was observed among incident cases, this points toward reverse causality in previous case‐control studies rather than a true inverse association.

Our stratified analyses by smoking status of incident cases showed that analysis among ever smokers only there was a suggestion of a protective effect of alcohol (*P*_trend_ = 0.07; Supplemental Table S1). This observation provides support for the hypothesis that residual confounding by smoking may have played a role in previous reports on the association between alcohol and PD.

Given the role of dopaminergic pathways in reward mechanisms, it has been hypothesized that PD patients might be less prone to addictive behaviors, either as a consequence of dopamine shortage or because of their genetic makeup.^18^ If alcohol consumption is indeed not associated with PD, the repeatedly suggested role of addictive behavior in predisposition to PD is not plausible, which offers further support to a true biological mechanism for components of cigarette smoke and coffee consumption in PD etiology.

A major strength of our analyses is that we had access to a large prospective cohort, with a mean follow‐up of 12.4 years^12^ and with lifetime lifestyle data (including alcohol consumption) collected at baseline. Furthermore, all PD cases were clinically confirmed by neurologists specialized in movement disorders,^12^ and by limiting our main analyses to incident cases, we circumvented any form of recall bias or reverse causation.

Observational studies on PD are complicated by the long prodromal phase of 20 years or more that can proceed the disease,^1^ although it is unclear if and how the nonmotor symptoms in this phase would affect alcohol consumption. Our stratified analyses by time to diagnosis indicated no association in either stratum.

Some exposure misclassification possibly occurred because we relied on self‐reported drinking habits. However, this possible misclassification would be nondifferential because we collected lifestyle information prospectively. Moreover, previous analyses within the EPIC cohort investigating alcohol consumption and other health outcomes have shown the information as of sufficient quality to detect known associations with cardiovascular and cancer outcomes.^14^, ^15^, ^16^ Furthermore, a clear and robust inverse association has been observed for smoking and PD risk within the NeuroEPIC4PD cohort,^3^ and no difference in misclassification between smoking and drinking habits is expected.

Different effects per beverage type have been suggested by some studies,^8^, ^9^ but not ours. Although our observations are in line with Palacios and colleagues,^10^ we might have missed possible effects for specific beverages as a result of exposure misclassification.

Alcohol consumption varied between countries (Supplemental Table S3), but a heterogeneity test (*P* = 0.71) indicated that associations between alcohol and PD risk were not different across countries. For part of the cohort, we had no data on lifetime alcohol consumption, which information was not available for Sweden and Naples (Italy). However, because there was no heterogeneity in effects between countries, these missing data will not have affected our findings.

Overall, our data support previous findings from large U.S. prospective studies that there is no association between alcohol consumption and the risk of PD.

## Disclaimer

Where authors are identified as personnel of the International Agency for Research on Cancer/World Health Organization, the authors alone are responsible for the views expressed in this article and they do not necessarily represent the decisions, policy, or views of the International Agency for Research on Cancer/World Health Organization.

## Author Roles

(1) Research project: A. Conception, B. Organization, C. Execution; (2) Statistical Analysis: A. Design, B. Execution, C. Review and Critique; (3) Manuscript: A. Writing of the first draft, B. Review and Critique.

S. Peters: 2A, 2B, 3A

V.G.: 1A, 1B, 1C, 3B

P.V.: 1A, 1B, 1C, 3B

L.T.M.: 1A, 1B, 1C, 3B

L.F.: 1A, 1B, 1C, 3B

C.S.: 1A, 1B, 1C, 3B

S.S.: 1A, 1B, 1C, 3B

A.K.: 1A, 1B, 1C, 3B

M.‐D.C.: 1C, 3B

R.Z.‐R.: 1C, 3B

O.H.: 1C, 3B

J.P.: 1C, 3B

V.K.: 1C, 3B

T.K.: 1C, 3B

O.M.: 1C, 3B

G.M.: 1C, 3B

E.A.: 1C, 3B

S. Panico: 1C, 3B

M.M.B.: 2C, 3B

T.J.K.: 1C, 3B

E.W.: 1C, 3B

P.F.: 1C, 3B

R.V.: 1A, 1B, 1C, 2C, 3B

**Financial disclosures of all authors (for the preceding 12 months):**

## Supporting information


**Appendix** **S1:** Supporting information
