# Supplementary material for: Alcohol Consumption and Risk of Parkinson's Disease: Data From a Large Prospective European Cohort
Source: Mov Disord. 2020 May 1;35(7):1258–63. doi: 10.1002/mds.28039 (PMC7496254; doi:10.1002/mds.28039)
Supplement: Supplementary file 1 — Appendix S1: Supporting information [file MDS-35-1258-s001.docx]

Supplemental Table 1. Number of PD cases and hazard ratios by average levels of lifetime alcohol consumption, stratified by smoking

| **Average lifetime alcohol consumption (g/day)** | **Never smokers** (n=100,475) | | **Ever smokers** (n=109,523) | |
| --- | --- | --- | --- | --- |
|  | PD cases | HR^1^ (95% CI) | PD cases | HR^1^ (95% CI) |
| Never consumer | 41 | 0.93 (0.63-1.35) | 7 | 0.93 (0.42-2.08) |
| 0.1-4.9 | 95 | 1.00 | 51 | 1.00 |
| 5.0-14.9 | 69 | 1.21 (0.87-1.67) | 73 | 1.31 (0.90-1.91) |
| 15-29.9 | 31 | 0.95 (0.59-1.51) | 46 | 1.19 (0.78-1.83) |
| 30-59.9 | 22 | 1.57 (0.91-2.68) | 25 | 0.75 (0.44-1.27) |
| ≥60 | 7 | 0.86 (0.37-1.98) | 11 | 0.68 (0.34-1.39) |
| P value for trend^2^ |  | 0.96 |  | 0.07 |

^1^Hazard ratio, adjusted for age at recruitment, sex, country and coffee consumption

^2^Trend among alcohol consumers only

Supplemental Table 2. Number of prevalent PD cases and hazard ratios by average levels of alcohol consumption

| **Alcohol consumption (g/day)** | **At recruitment** | | **Lifetime** | |
| --- | --- | --- | --- | --- |
|  | PD cases (n=92) | HR^1^ (95% CI) | PD cases (n=73) | HR^1^ (95% CI) |
| Non-/Never consumer | 22 | 1.25 (0.71-2.23) | 9 | 1.60 (0.74-3.47) |
| 0.1-4.9 | 29 | 1.00 | 29 | 1.00 |
| 5.0-14.9 | 23 | 0.86 (0.49-1.48) | 25 | 0.77 (0.45-1.35) |
| 15-29.9 | 10 | 0.79 (0.38-1.65) | 7 | 0.48 (0.20-1.15) |
| 30-59.9 | 5 | 0.69 (0.26-1.84) | 3 | 0.36 (0.10-1.28) |
| ≥60 | 3 | 1.59 (0.45-5.54) | 0 | - |
| P value for trend^2^ |  | 0.93 |  | 0.04 |

^1^Hazard ratio, adjusted for age at recruitment, sex, country and coffee consumption

^2^Trend among alcohol consumers only

Supplemental Table 3. Demographics and alcohol consumption habits among NeuroEPIC4PD participants, by country

|  | **Italy** | **Spain** | **UK** | **NL** | **Greece** | **Germany** | **Sweden** |  |
| --- | --- | --- | --- | --- | --- | --- | --- | --- |
|  | N=40,175 | N=24,957 | N=23,398 | N=16,827 | N=25,854 | N=25,439 | N=53,348 |  |
| **Sex** |  |  |  |  |  |  |  |  |
| Male | 11,436, 28% | 10,724, 43% | 10,571, 45% | - | 10,586 41% | 11,875, 47% | 23,203, 43% |  |
| Female | 28,739, 72% | 14,233, 57% | 12,827, 55% | 16,827, 100% | 15,268, 59% | 13,564, 53% | 30,145, 57% |  |
| **Age at recruitment**, mean | 51.1 | 49.3 | 59.4 | 57.7 | 53.4 | 50.9 | 52.3 |  |
| **Alcohol consumption at recruitment** | |  |  |  |  |  |  |  |
| Non-consumer | 6,739, 17% | 7,228, 29% | 4,769, 20% | 3,090, 18% | 6,667, 26% | 1,621, 6% | 7,698, 14% |  |
| Total g/day, mean | 14.2 | 16.0 | 8.17 | 9.01 | 9.53 | 17.8 | 6.81 |  |
| Beer g/day, mean | 0.84 | 1.67 | 2.28 | 0.52 | 2.03 | 7.85 | 2.24 |  |
| Wine g/day, mean | 12.0 | 12.5 | 3.91 | 4.50 | 4.83 | 9.08 | 2.36 |  |
| Fortified wine g/day, mean | 0.44 | 0.18 | 0.56 | 2.44 | 0.01 | 0.10 | 0.85 |  |
| Spirits g/day, mean | 0.89 | 1.70 | 1.42 | 1.55 | 2.67 | 0.77 | 1.37 |  |
| **Average lifetime alcohol consumption^a^** | |  |  |  |  |  |  |  |
| Never consumer | 4,418, 13% | 4,172, 17% | 1,258, 6% | 2,037, 12% | 5,755, 22% | 600, 2% | - |  |
| Total g/day, mean | 14.1 | 26.8 | 10.3 | 8.12 | 18.6 | 17.8 | - |  |
| Beer g/day, mean | 0.78 | 3.10 | 3.05 | 0.59 | 2.70 | 9.59 | - |  |
| Wine g/day, mean | 12.1 | 18.7 | 3.00 | 3.79 | 8.16 | 5.15 | - |  |
| Fortified wine g/day, mean | 0.21 | 0.83 | 0.68 | 2.22 | 1.29 | 0.31 | - |  |
| Spirits g/day, mean | 1.06 | 4.16 | 3.59 | 1.52 | 6.48 | 2.76 | - |  |
| **Smoking status at recruitment** | |  |  |  |  |  |  |  |
| Never | 18,613, 46% | 13,160, 53% | 10,704, 45% | 7,230, 43% | 14,416, 56% | 10,651, 42% | 25,701, 48% |  |
| Former | 10,649, 27% | 4,523, 18% | 9,343, 40% | 5,820, 35% | 4,338, 17% | 8,854, 35% | 14,591, 27% |  |
| Current | 10,913, 27% | 7,274, 29% | 3,351, 14% | 3,777, 22% | 7,100, 27% | 5,934, 23% | 13,056, 24% |  |
| **Coffee consumption at recruitment** | |  |  |  |  |  |  |  |
| Non-consumer | 3,618, 9% | 2,635, 11% | 2,782, 12% | 466, 3% | 1,685, 7% | 1,280, 5% | 2,113, 4% |  |
| >0 to <100 mL/day | 17,882, 45% | 10,284, 41% | 2,761, 12% | 345, 2% | 7,273, 28% | 2,912, 11% | 2,257, 4% |  |
| 100 to <250 mL/day | 17,366, 43% | 8,997, 36% | 5,018, 21% | 617, 4% | 11,522, 45% | 2,968, 12% | 7,444, 14% |  |
| 250 to <500 mL/day | 1,253, 3% | 2,703, 11% | 7,376, 32% | 4,159, 25% | 3,908, 15% | 9,686, 38% | 19,561, 37% |  |
| ≥500 mL/day | 56, 17% | 338, 1% | 5,461, 23% | 11,240, 67% | 1,466, 6% | 8,593, 34% | 21,973, 41% |  |

^a^Information on lifetime alcohol consumption was missing for 216 PD cases and 59,585 participants without PD
